# Biomechanical evidence for occupational specialization in Mesolithic-Neolithic hunter-gatherers from Zvejnieki, Latvia

**DOI:** 10.1126/sciadv.aed3371

**Published:** 2026-06-12

**Authors:** Daniel H. Temple, Gunita Zariņa, Ilga Zagorska, Aija Macāne

**Affiliations:** ^1^ Department of Sociology and Anthropology, George Mason University, Fairfax, VA, USA.; ^2^ Institute of Latvian History, Faculty of Humanities, University of Latvia, Riga, Latvia.; ^3^ Department of Cultures, University of Helsinki, Helinski, Finland.

## Abstract

Mesolithic cemeteries have greater dimensionality and scale that reflect emerging social complexity following the Upper Paleolithic. The Zvejnieki cemetery dates between the Middle Mesolithic and Late Neolithic, is associated with a persistent hunter-fisher-gatherer economy, and includes burials with pendants fashioned from mammal teeth. Stable isotopic analyses indicate dietary differences between individuals with pendants and without pendants beginning early in life. This context provides an opportunity to explore questions relating to occupational specialization using long bone cross-sectional geometry. Femoral cross-sectional properties from Zvejnieki were compared to a European database from the Upper Paleolithic to Neolithic to explore mobility. Femoral and humeral cross-sectional properties were compared between individuals with and without pendants to understand differences in mobility and manual activity in relation to mortuary practices. Diminished femoral rigidity and circular femoral shape were found at Zvejnieki, but no differences were observed between burial groups. Expanded cortical area and diminished medullary area were observed in the right humeri of males with pendants. Right humeral shape was comparatively circular among males with pendants, while right and left humeral shape was anteroposteriorally reinforced among males without pendants. Females with pendants had elevated right and left humeral shape ratios compared to females without pendants. Femoral morphology reflects reduced mobility, interaction with flat terrain, and exploitation of abundant, local resources at Zvejnieki. Differences in humeral cross-sectional properties between burial groups are consistent with specialized manual activity. These patterns reflect the economic and ideological components of independent specialization and point toward expanding social dimensions in Mesolithic-Neolithic hunter-gatherers.

## Teaser

Biomechanics reveal specialized manual activity linked to mortuary practices and diet in Mesolithic-Neolithic hunter-gatherers.

## INTRODUCTION

The European Mesolithic represents a transformative socioecological period that followed the Upper Paleolithic and preceded settled agricultural life in the Neolithic (*1*, *2*). Expansion of boreal and oak forests combined with retreating ice sheets beginning in the Late Glacial Interstadial opened up ecological niches that supported large-scale exploitation of localized resources (*1*, *2*). Mesolithic communities increased in size and decreased in mobility over time and in relation to the acquisition of abundant, defensible resources (*2*), although important cultural and ecological contingencies in these patterns are noted (*3*, *4*). Large cemeteries with elaborate grave goods and body preparation are documented in the Mesolithic (*2*, *5*–*8*) and represent a substantial increase in dimensionality and scale when compared with Late Upper Paleolithic funerary behavior (*2*, *9*–*12*). Increased dimensionality and scale of funerary behavior are associated with the exploitation of abundant and defensible local resources (*5*, *7*, *13*–*15*). Elaborate distributions of grave goods and spatial patterning within these cemeteries reflect increases in social complexity and have led to deeper speculation regarding the emergence of institutional inequality, occupational specialization, and broader relationships between local cosmologies and mortuary rites (*5*–*8*, *13*–*16*).

Complexity refers to increases in the number of interrelated parts and internal differentiations within the social organization of communities (*17*). Occupational specialization is an integral component of social complexity that is defined by the organization of production: Craft specialists transform raw materials into usable objects, and livelihoods are sustained through the temporally durable manufacture and production of these items (*18*, *19*). Archaeological research on occupational specialization often focuses on the production of specialized material goods and spatial analyses that explore the distribution of locations within sites where specialized items are produced (*20*). These behaviors are often emphasized in food-producing societies (*17*–*21*). However, the production of specialized material goods and spatial analysis that identify discrete site locations where these goods are produced indicate the presence of occupational specialization in Holocene hunter-gatherers (*22*–*28*). Less is known regarding the emergence of occupational specialization as a social dimension of mortuary practices in Mesolithic-Neolithic hunter-gatherers. This work explores embodied evidence for occupational specialization as a social dimension of mortuary practices in Mesolithic-Neolithic hunter-gatherers and endeavors to understand these behaviors in relation to mobility. Results will identify underlying complexities in Mesolithic-Neolithic hunter-gatherer behavior and the broader ecological and social contexts associated with the emergence of these social dimensions in relation to mortuary practices.

Human skeletal remains provide embodied evidence for habitual activity, and archaeological mortuary practices track dimensions of social organization and concurrent ideological behavior (*29*, *30*). Long bone diaphyses are structurally responsive to strain levels experienced during the life course: Bone experiences periosteal expansion and later endosteal deposition in response to mechanical demands during ontogeny and modifies or maintains these properties in response to loading patterns in adulthood (*31*, *32*). Cross-sectional geometric properties may be used to quantify the response of long bone diaphyses to changes in mechanical loading (*33*). Cross-sectional properties included in this work are documented in Table 1. Cross-sectional properties were drawn from the anteroposterior (A-P) (*I_x_*) and mediolateral (M-L) (*I_y_*) rigidity of the humerus and femur, overall rigidity (*J*) of the humerus and femur, and shape ratios of A-P relative to M-L rigidity (*I_x_*/*I_y_*) of the humerus and femur. In addition, owing to complexities in interpreting diaphyseal loading in the upper limb (*34*), humeral cross-sectional properties also include total subperiosteal area (TA), total cortical area (CA), and medullary area (MA).

**Table 1.** Cross-sectional properties. Abbreviations and definitions for cross-sectional properties included in this study.

Property	Abbreviation	Units	Definition
Total subperiosteal area	TA	mm^2^	
Cortical area	CA	mm^2^	Axial rigidity
Medullary area	MA	mm^2^	
Second moment of area about the *x* axis	*I_x_*	mm^4^	A-P bending rigidity
Second moment of area about the *y* axis	*I_y_*	mm^4^	M-L bending rigidity
Polar second moment of area	*J*	mm^4^	Torsional/twice bending rigidity

Studies of femoral diaphyseal morphology identify diminished bending *J* and *I_x_*/*I_y_* with the advent of agriculture that reflects reductions in overall mobility (*35*, *36*). In addition, femoral *I_x_*/*I_y_* is associated with terrain interaction, specifically changes in *I_x_* which increases in response to more complex terrain and reduces in response to flatter terrain (*37*, *38*). Humeral diaphyseal morphology has the capacity to reveal intensity and patterning of manual activity (*33*, *39*). Bilateral increases in *J* and reductions of humeral *I_x_*/*I_y_* (≤1.00) are often associated with forearm strengthening, possibly in association with rowing (*34*, *40*), while elevated asymmetry in overall humeral rigidity combined with a circular shape (*I_x_*/*I_y_* ∼ 1.0) are associated with torsional loading related to throwing (*40*–*43*). More recently, bilateral increases in *I_x_*/*I_y_* were associated with the mechanical demands of flexion and extension of the forearm, which was interpreted within the context of archaeological, experimental, and ethnographic depictions of hide processing (*40*, *44*, *45*). Unilateral increases in the same properties may be attributable to spear thrusting or hide processing (*44*, *46*). Moments of area may further reveal the processes contributing to differences in skeletal strength and rigidity: This includes patterns of surface deposition that expand the external perimeter of bone and accentuate diaphyseal strength or endosteal bone deposition and medullary contraction which has limited impact on diaphyseal strength (*31*, *47*).

Evidence for sexual divisions of labor, temporal changes in manual activity, and unique humeral morphology in relation to isolated burial practices are reported in the Upper Paleolithic (*36*, *48*, *49*). Studies of Mesolithic cemeteries document geographic and temporal variation in behavior. However, few studies have attempted to link cross-sectional properties to mortuary practices and diet within cemeteries (*50*). In one instance, limited evidence for differences in mobility and manual activity was documented between burial groups at Yuzhni Olenni’ ostrov in Northern Eurasia (*51*). These findings suggest that cross-sectional geometric analyses of long bone diaphyses have great potential to help reconstruct behaviors related to occupational specialization, expand dimensionality of Mesolithic mortuary practices, and document mobility patterns associated with the emergence of social complexity.

**Fig. 1.**
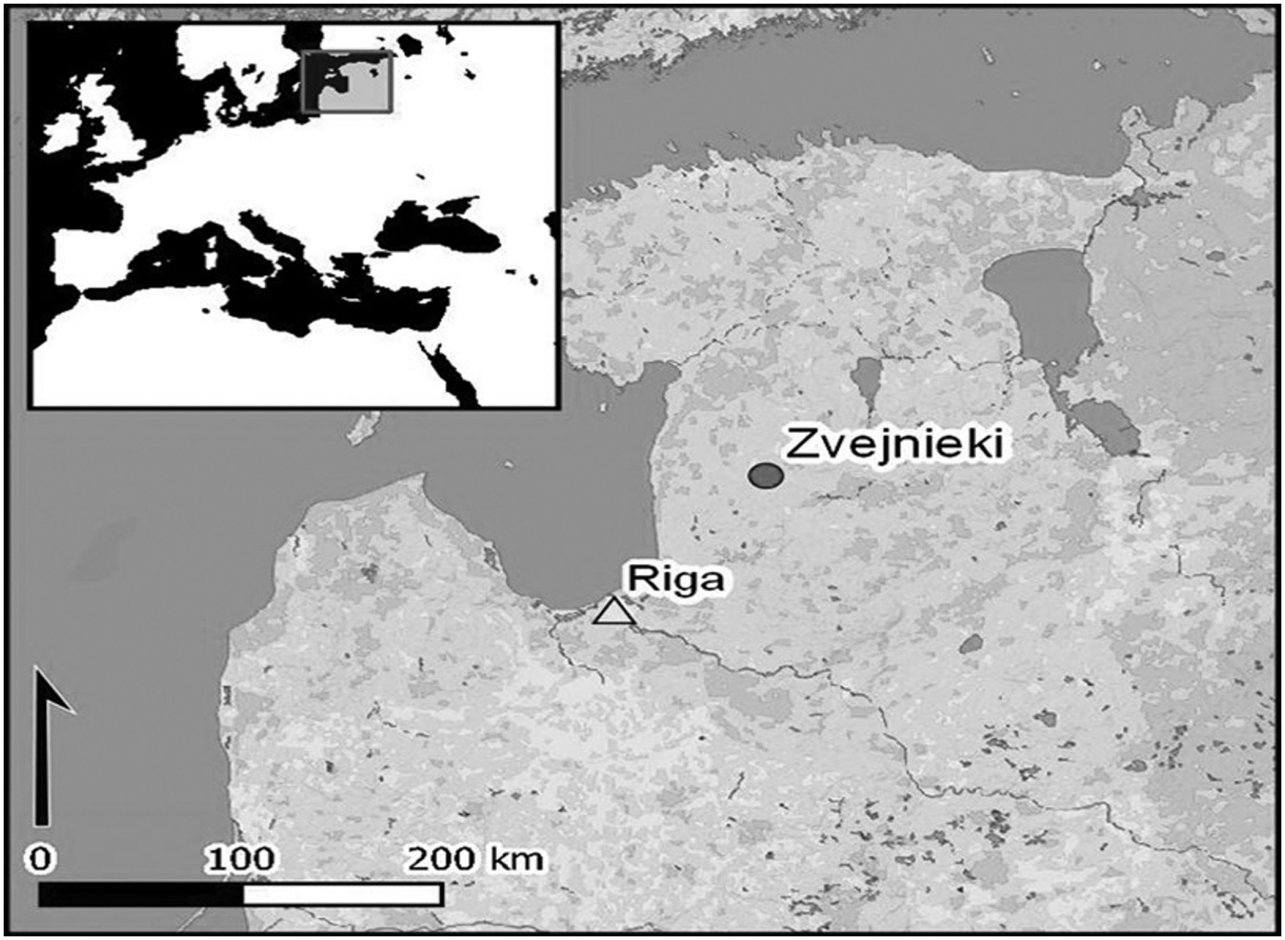
Site map. Location of the Zvejnieki site in northern Latvia.

This study focuses on human remains from the Zvejnieki site. Zvejnieki is located in northern Latvia on the northern shore of Lake Burtnieks, a freshwater lake that receives upflow from approximately seven streams (Fig. 1). Radiocarbon chronologies and cultural affiliations date the remains included in this study between the Middle Mesolithic (seventh millennium BCE) and Late Neolithic (fourth millennium BCE) (*6*, *52*). This time period is characterized by a sustained hunter-fisher-gatherer economy as farming did not begin in this region until the third and second millennium BCE (*6*). These time periods occur across two climatic phases of the Blytt-Sernander sequence including the Atlantic (7500–5000 cal. BCE) and Subboreal (5000–2500 cal. BCE) (*53*, *54*). Material culture, zooarchaeological remains, and stable isotope analysis suggest intensive exploitation of anadromous fish from the lake combined with hunting of terrestrial mammals (*15*, *55*–*57*). Evidence for differences in the incorporation of these foods into the diet across the Mesolithic-Neolithic boundary is noted, although the persistence of a hunter-fisher-gatherer subsistence spectrum is recognized through the advent of Comb Wear pottery (*15*, *55*, *56*, *58*). A study of long bone cross-sectional geometry at the site found little evidence for temporal change in mobility, terrain interaction, or manual activity between Mesolithic and Neolithic occupations (*59*).

Approximately 330 burials containing at least 350 individuals have been excavated from Zvejnieki (*6*, *52*, *60*). Mortuary practices at the site include diverse grave goods such as bone and stone tools, animal remains, ornaments made from animal remains, amber ornaments, anthropomorphic figurines, and pottery (*6*, *52*, *60*), as well as close spatial affiliations between the Mesolithic and Neolithic burials (*52*, *61*). Approximately 98 burials are securely associated with pendants fashioned from animal teeth (*6*, *60*). These pendants were drilled, cut, or grooved and attached to garments or strung together (*6*). Pendants are found in the graves of preadults and adults as well as individuals estimated to be female and male from the Middle Mesolithic to Late Neolithic periods (*6*, *60*). While many pendants were worn as adornments in life, use-wear analysis suggests that a small subset of these items may have used exclusively in burial (*62*). Pendants include teeth from numerous herbivorous ungulate and carnivore species: Herbivorous ungulates dominate burial assemblages through 5000 cal. BCE, when carnivore species appear and increase in frequency (*60*). This shift is concomitant with the Atlantic/Subboreal transition, which likely influenced the ecological and social spheres of interaction between humans and terrestrial fauna (*60*).

Recent isotopic studies found reduced nitrogen values among individuals buried with pendants and elevated nitrogen values among individuals without pendants (*58*). These results are consistent with greater consumption of terrestrial mammals among adult individuals with pendants and greater consumption of freshwater resources among adult individuals buried without pendants. Isotopes derived from incremental sequences of dentin found greater consumption of freshwater resources in the postweaning diet of individuals without pendants and greater consumption of terrestrial mammals in the postweaning diet of individuals with pendants across the Mesolithic to Middle Neolithic occupation (*56*, *58*). These patterns of dietary variation are hypothesized to reflect differential access to resources in association with occupational specialization (hunting terrestrial mammals versus exploitation of aquatic resources) and familial identities related to these specializations (*58*). The exploration of long bone cross-sectional geometry in relation to these burials provides an opportunity to test the general hypothesis that occupational specialization structured variation in diet and mortuary practices and that these behaviors were supported by a localized, more permanent settlement pattern. Here, three subhypotheses relating cross-sectional geometry and mortuary practices to occupational specialization and mobility are tested: (i) Individuals with burial pendants will have elevated levels of femoral rigidity in relation to hunting terrestrial mammals across greater distances and more diverse terrain; (ii) femoral rigidity as well as shape indices will be reduced at Zvejnieki compared with European Paleolithic and Mesolithic hunter-gatherers due to reductions in mobility and interaction with flatter terrain; (iii) differences in directional asymmetry, humeral rigidity, and humeral shape indices will be observed between individuals with and without pendants and indicate differences in the intensity and pattern of activity in the upper limb.

## RESULTS

Sample sizes included in this study are reduced as many burials were preadults (30%), few were adult females (15%) (*6*), and this work focuses on adult cross-sectional geometry (Tables 2 and 3)—infants, children, and adolescents have unique patterns of bone deposition between ages that is hormonally mediated (*31*, *47*, *63*, *64*). In addition, only individuals with isotopic values were included to maintain consistency with dietary analyses. Comparisons between females with and without pendants used 95% credibility intervals between groups, while comparisons of combined female burial groups (pendant and nonpendant burials) from Zvejnieki to females across different time periods used 95% credibility intervals and randomized distributions of mean differences. All male comparisons relied on 95% credibility intervals and randomized distributions of mean differences. This approach carries fewer distributional assumptions and provides direct estimates of effect size and uncertainty (see Materials and Methods).

**Table 2.** Sample composition. Sample composition of femoral cross-sectional analysis for the Zvejnieki and comparative groups.

Group	*N* Female femora	*N* Male femora
Early Upper Paleolithic	5	9
Late Upper Paleolithic	7	14
Mesolithic	14	36
Neolithic	106	155
Zvejnieki Pendant	2	15
Zvejnieki None	5	19

**Table 3.** Sample composition. Sample composition of humeral cross-sectional analysis for the Zvejnieki and comparative groups.

Group	*N* Right humeri	*N* Left humeri	*N* Left + right humeri
Zvejnieki pendant female	1	3	1
Zvejnieki no pendant female	5	6	5
Zvejnieki pendant male	13	9	6
Zvejnieki no pendant male	35	23	28

**Fig. 2.**
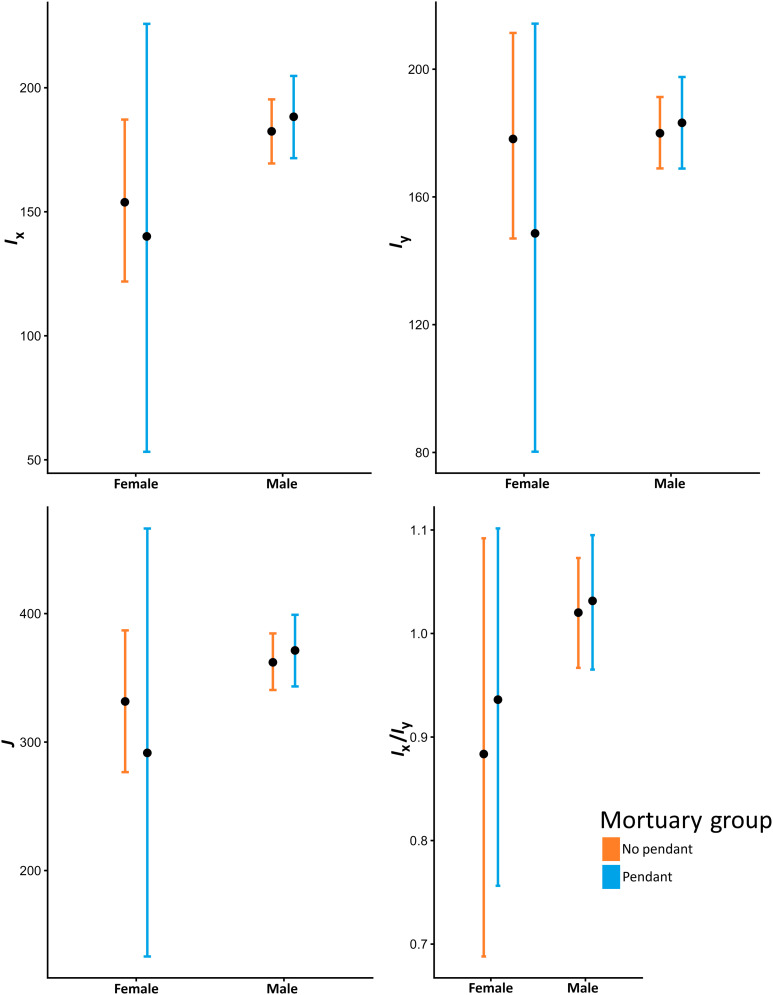
95% Credibility intervals for Zvejnieki femora. Individuals with pendants are indicated in blue, while individuals without pendants are indicated in orange. Group means are indicated by a solid circle. Sex is indicated on the *x* axis.

Overlap in the 95% credibility intervals for females and males with and without pendants is observed (Fig. 2). The observed mean differences (∆) of femoral cross-sectional properties for males with and without pendants are distributed within the region of practical equivalence (ROPE) for *I_x_* (∆=−12.48;ROPE=−18.23−18.23), *I_y_* (∆=−9.31;ROPE=−17.97−17.97), *J* (∆=−21.80;ROPE=−36.20−36.20), and *I_x_*/*I_y_* (∆=−0.019;ROPE=−0.031−0.031) further indicating comparability between burial groups.

**Fig. 3.**
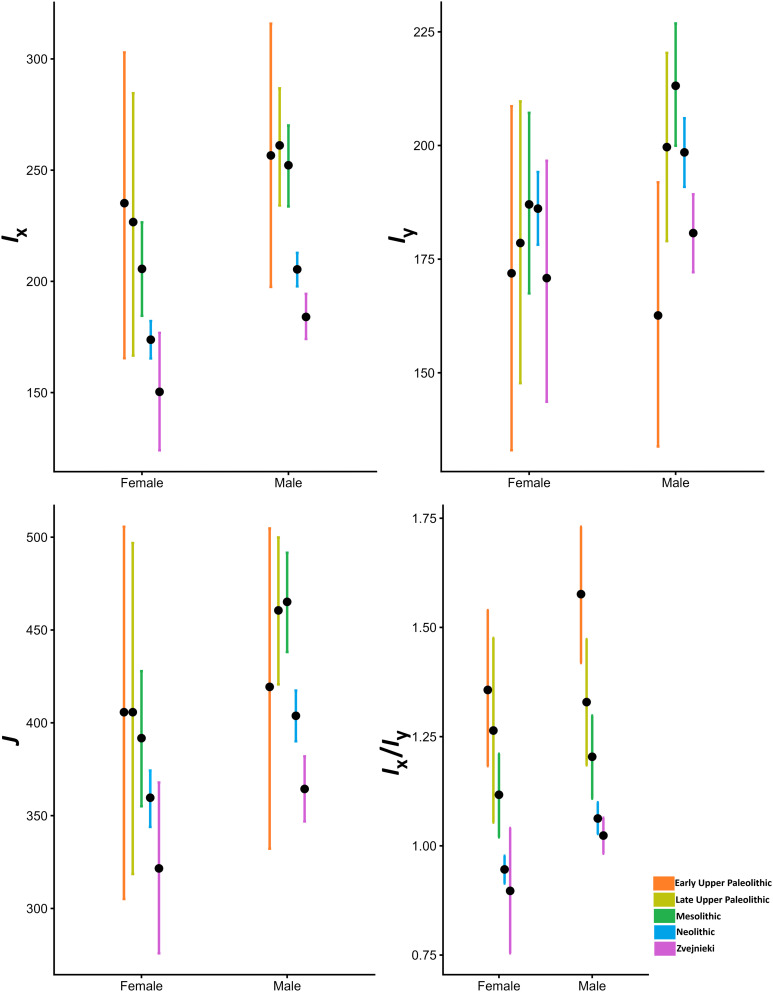
95% Credibility intervals for European femora. Zvejnieki (purple) relative to Early Upper Paleolithic (orange), Late Upper Paleolithic (yellow), Mesolithic (green), and Neolithic (blue) communities, with group means indicated by a solid circle. Sex is indicated on the *x* axis.

**Fig. 4.**
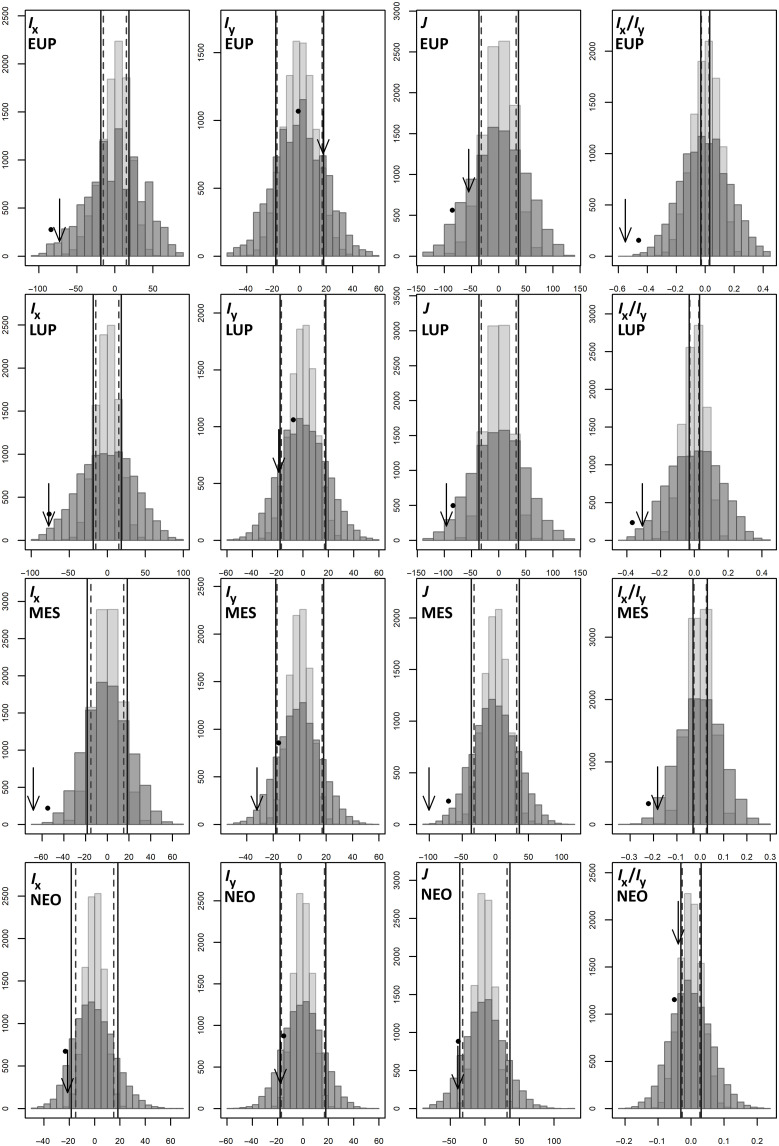
Randomized permutations of mean differences in femoral cross-sectional properties. Zvejnieki compared to Early Upper Paleolithic (EUP), Late Upper Paleolithic (LUP), Mesolithic (MES), and Neolithic (NEO) groups. Randomized distributions of female mean differences are the dark-shaded bars, with ROPEs between the dashed lines. Randomized distributions of male mean differences are the light-shaded bars, with ROPEs between the solid lines. Female observed mean differences are indicated by the circle, and male observed mean differences are indicated by the arrow within each distribution.

The 95% credibility intervals document a sustained reduction in femoral *I_x_*, *J*, and *I_x_*/*I_y_* between the Early Upper Paleolithic and Neolithic (Fig. 3) that is consistent with reduced mobility and interaction with complex terrain as communities moved toward settled agricultural economies (*38*, *65*). The 95% credibility intervals indicate greater similarity between Zvejnieki females and males with settled Neolithic communities for *I_x_*, *I_y_*, and *J*. These same values are reduced when Zvejnieki females and males are compared with Paleolithic and Mesolithic hunter-gatherers. Observed mean differences between females from Zvejnieki compared with Early Upper Paleolithic, Late Upper Paleolithic, and Mesolithic females are reduced and distributed outside the ROPEs for *I_x_*, *J*, and *I_x_*/*I_y_* (Fig. 4). Observed mean differences between males from Zvejnieki compared with Early Upper Paleolithic, Late Upper Paleolithic, and Mesolithic males are reduced and distributed outside the ROPEs for *I_x_*, *J*, and *I_x_*/*I_y_* and within or near the boundary of the ROPEs for *I_y_* (Fig. 4). In contrast, observed mean differences for females and males from Zvejnieki compared with females and males from Neolithic communities are reduced but distributed near or within the ROPEs for all cross-sectional variables (Fig. 4).

**Fig. 5.**
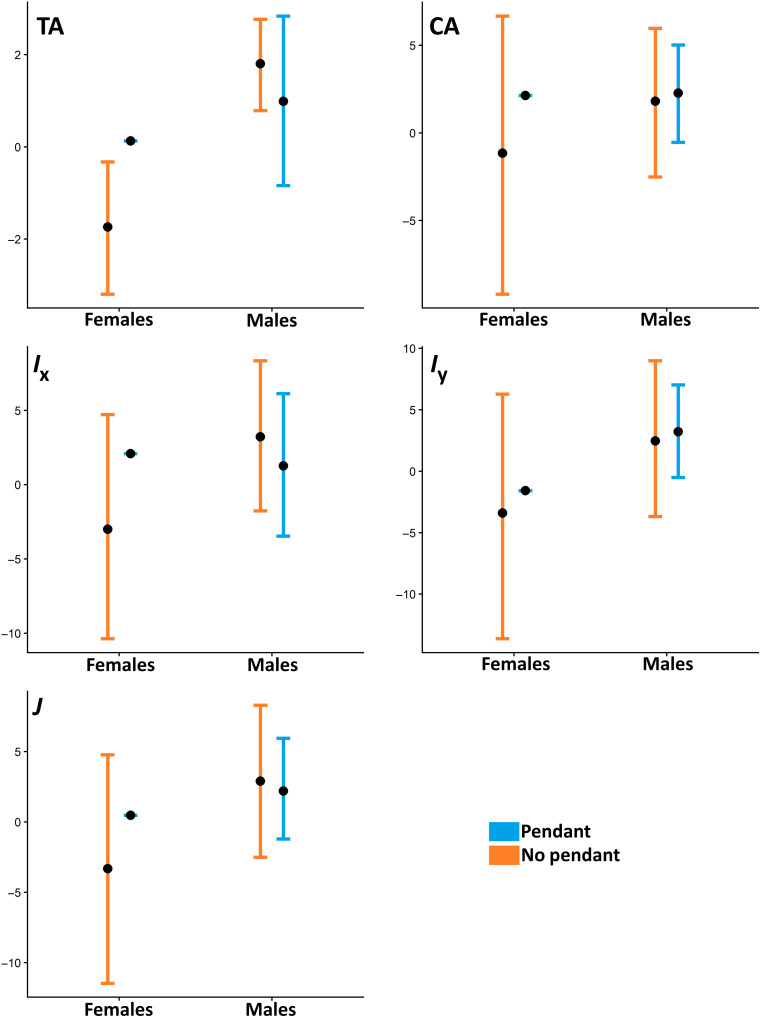
95% Credibility intervals of directional asymmetry for humeral cross-sectional properties. Females and males with pendants are indicated in blue, and females and males without pendants are listed in orange, with group means indicated by a solid circle. Sex is listed on the *x* axis.

The 95% credibility intervals for humeral directional asymmetry for females and males with and without pendants respectively overlap for each cross-sectional geometric property (Fig. 5). In addition, observed mean differences for males with and without pedants are distributed within all ROPEs for TA (∆=−0.39%), CA (∆=0.87%), *I_x_* (∆=−0.95%), *I_y_* (∆=0.98%), and *J* (∆=−0.01%).

**Fig. 6.**
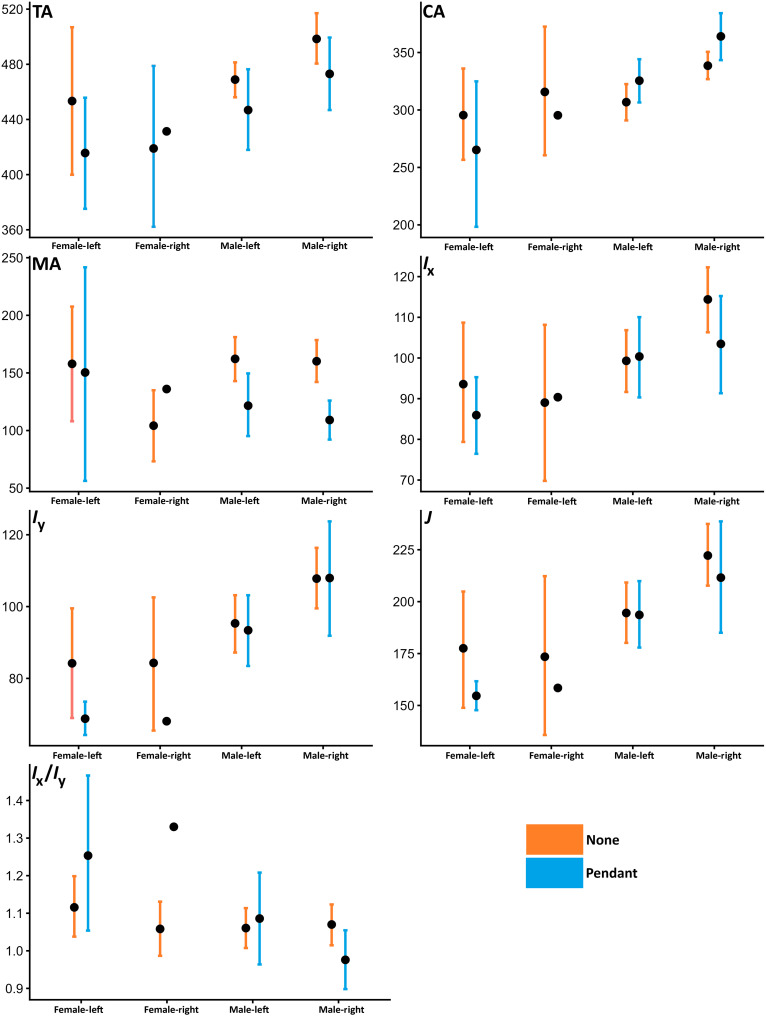
95% Credibility intervals of cross-sectional properties for left and right humeri. Females and males with pendants are listed in blue; females and males without pendants are listed in orange; group means are indicated by a solid circle. Sex and side are listed on the *x* axis.

**Fig. 7.**
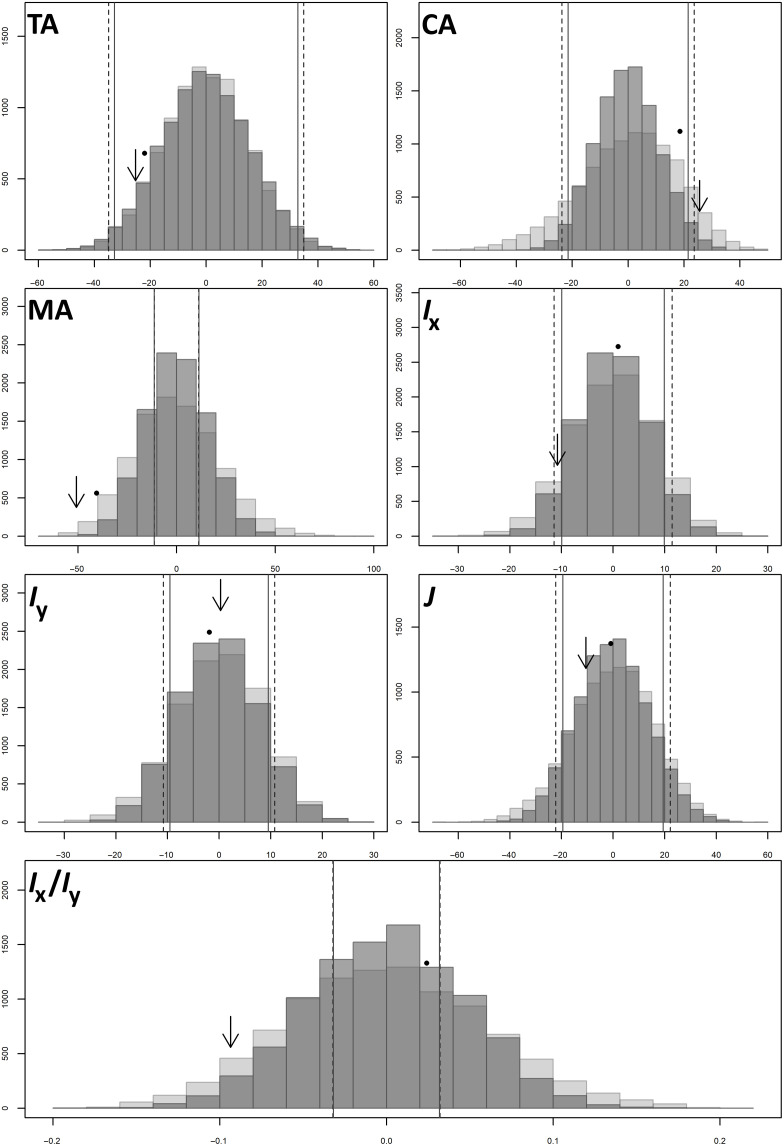
Randomized permutations of mean differences in humeral cross-sectional properties for Zvejnieki males. Randomized distributions of mean differences for right humeral properties are indicated by light-shaded bars, with ROPEs between the solid lines. The observed mean differences for right humeral properties are indicated with an arrow in each distribution. Randomized distributions of mean differences for left humeral cross-sectional properties are indicated by dark-shaded bars, with the ROPEs located between the dashed lines. The observed mean differences for left humeral cross-sectional properties are indicated by a circle within each distribution.

Overlap in the 95% credibility intervals is observed for all left humeral cross-sectional properties in males (Fig. 6). All left humeral cross-sectional properties for males are also distributed within the ROPE, excepting MA which has limited standalone interpretive value (Fig. 7). Right humeral CA is elevated, while right humeral MA is reduced among males with compared to without pendants (Fig. 6). Mean differences for both variables are distributed outside the ROPE (Fig. 7). Minimal overlap is also observed in 95% credibility intervals for right humeral *I_x_*/*I_y_*: Males with pendants have reduced humeral *I_x_*/*I_y_* with distributions that overlap with zero, while males without pendants have larger *I_x_*/*I_y_* that do not overlap with zero (Fig. 6). The observed mean difference of male humeral *I_x_*/*I_y_* between individuals with pendants and without pendants is outside the ROPE for the randomized distribution of mean differences (Fig. 7). This is associated with reduced right humeral *I_x_* and comparable right humeral *I_y_* in males with pendants versus without pendants (Figs. 6 and 7). Elevated left and right humeral *I_x_*/*I_y_* is observed among females with pendants compared to those without pendants (Fig. 6). This is associated with comparable left and right humeral *I_x_* but reduced humeral *I_y_* among females with compared to those without pendants (Fig. 6). Females without pendants have elevated *I_x_*/*I_y_* values (overlap with 1.0 is negligible) for left and right humeri, although these values remain reduced compared to females with pendants.

## DISCUSSION

The first hypothesis of this study was not supported. Femoral *I_x_*, *I_y_*, *J*, and *I_x_*/*I_y_* values do not differ between burials with pendants and those without pendants for females or males. This differs from previous studies that report slight differences in femoral head diameter and midshaft circumference between burial classes at Yuzhniy Olenni ostrov (*51*). This may be attributable to the use of peripheral quantitative computed tomography (PQCT) scanning techniques that provide more reliable cross-sectional estimations. In addition, at Zvejnieki, the lack of differences in femoral robusticity between burial groups may be attributable to the generally reduced pattern of mobility and flatter terrain around the site. Here, the second hypothesis of the study is supported. Zvejnieki female and male femora were less robust and more circular than Upper Paleolithic and Mesolithic femora, while cross-sectional properties were comparable to Neolithic femora. This finding is generally consistent with previous studies that report limited femoral diaphyseal robusticity around the Baltic region during the Mesolithic-Iron Age but especially among hunter-gatherer communities (*59*). At Zvejnieki, femoral *I_x_*, *J*, and *I_x_*/*I_y_* are reduced, but comparable *I_y_* values are documented in comparison to European Paleolithic and Mesolithic groups. These results are indicative of interaction with flat terrain and reduced mobility compared with other hunter-gatherer communities (*37*, *38*). Zvejnieki is located along the shores of Lake Burtnieks and was surrounded by woodlands dominated by *Pinus* and broad-leaf deciduous species during the Boreal to Subboreal chronozones (*55*). Fish and terrestrial mammals were sourced from these ecosystems (*15*, *55*, *58*, *60*). The terrain surrounding Zvejnieki is also relatively flat; relief maps of the Baltic Sea region indicate that the largest hills within 50 km of Zvejnieki are 121 m, and Zvejnieki femora are less robust than hunter-gatherers from regions with documented complexity in terrain (*66*). In this sense, the local environment at Zvejnieki likely facilitated proximate access to abundant lacustrine and terrestrial resources, with limited exposure to complex terrain. These findings are consistent with the general hypothesis that European Mesolithic landscapes associated with abundant access to resources and increasing dimensionality and scale in mortuary practices facilitated reductions in mobility (*5*, *13*).

The third hypothesis of this study garners partial support. No differences in right or left directional asymmetry are found between female or male burials with pendants and without pendants. Average directional asymmetry is also low in these groups (*67*). Reduced humeral strength and asymmetry are associated with the introduction of bow and arrow as well as net fishing technologies (*40*, *41*, *68*, *69*). Bow and arrow as well as net fishing usage may have diminished the intensity of unimanual projectile weapon use at Zvejnieki, particularly following the Late Mesolithic (*70*). However, right humeral CA is elevated, while right humeral MA is reduced among males with pendants versus without pendants. These results are consistent with greater endosteal deposition of cortical bone in the right humeri of males with pendants compared to without pendants. Similar results are observed in the playing arm of semi-elite adolescent athletes, where unimanual bone strain resulted in cortical deposition on the endosteum and medullary contraction (*47*, *71*). This pattern of variation is associated with hormonal shifts in adolescence that redirect skeletal deposition to the endosteal surface in response to intense or repetitive strain (*31*, *47*, *64*). In cases of throwing, cortical area and bone mineral content are expanded in the distal humerus (*32*, *72*). These findings indicate a pattern of right-dominant diaphyseal loading, likely beginning in adolescence, among males with pendants versus without pendants.

In addition, right humeral *I_x_*/*I_y_* is reduced and comparatively circular among males with pendants versus those without pendants. This difference in humeral shape is associated with smaller *I_x_* and comparable *I_y_* values among males with pendants compared to without pendants. While humeral shape ratios are complex in interpretation, careful understandings of bone loading dynamics and the archaeological record may help reveal potential contributors to this variation. Throwing increases torsional loading of the humeral shaft. Torsional loading that results from throwing is resisted across A-P and M-L axes in the distal humerus, and bone deposition is greatest in these planes (*32*, *72*, *73*). This pattern of strain produces a more circular humeral shape and is found in the dominant arms of clinical studies of cricket bowlers, baseball pitchers, and following the evolution of unimanual projectile weapons (*32*, *43*, *72*, *74*). Hunting technologies at Zvejnieki include unimanual projectiles such as bone spears, spearheads, and arrowheads as well as bone harpoons—used for the procurement of lacustrine and terrestrial prey (*75*, *76*). Given the diverse array of projectile hunting technology uncovered at Zvejnieki, it is not possible to attribute these behaviors to a specific projectile weapon or prey choice. However, the results identify distinct humeral loading patterns among individuals buried with pendants that are consistent with greater usage of unimanual projectile weapons. Given the correspondence with dietary differences and mortuary practices (*58*), it is reasonable to surmise that these activities afforded greater access to foodstuffs derived from terrestrial mammals.

Elevated bimanual humeral shape ratios are found in males without pendants compared to those with pendants. This is associated with elevated *I_x_* values on the right and similar *I_x_* values on the left sides when individuals without pendants and with pendants are compared. Flexion and extension of the elbow is associated with increased humeral *I_x_* through the actions of biceps and triceps muscles (*77*). Elevated *I_x_*/*I_y_* are identified in communities where net fishing was common and is contrasted with unimanual circular shape ratios in groups where throwing was emphasized (*40*, *78*). One potential reason for elevated bimanual *I_x_*/*I_y_* in relation to net fishing is flexion and extension during the lifting and pulling of baskets, lines, and nets (*40*, *78*). Fishing technologies at Zvejnieki during the Late Mesolithic and Early Neolithic included hooks, knives, sinkers, and baskets (*70*). The adoption of these technologies at Zvejnieki is consistent with a specialized pattern of habitual activity that produced increased A-P loading among males without pendants.

Female humeral sample sizes are reduced. However, several interesting trends in the distribution of these cross-sectional data are found. Accentuated left and right humeral *I_x_*/*I_y_* is observed in females with pendants. This is associated with reduced *I_y_* in females with pendants compared to without pendants. This finding indicates reduced strain in the M-L plane and concentrated habitual activity in the A-P plane of both humeri. Experimental work documents increased maximum and total muscle activity for pectoralis major and anterior deltoideus during hide processing, which may result in increased A-P reinforcement of right and left humeri (*44*). Bioarchaeological studies report accentuated *I_x_*/*I_y_* in groups where hide processing is documented (*40*, *45*). At Zvejnieki, axes are ubiquitously distributed at the settlement (*79*) and found in select female graves, where each grave with an axe also included pendants (five total burials, 5.1% of all pendant burials: three adolescent-adult aged females, two multiple burials that include adult-aged females) (*6*, *79*). A high-resolution analysis of one funerary axe from the site found wear patterns suggesting repetitive A-P dominant movement, ochre concentrated in the axe, and polishing consistent with exposure to animal lard (*79*). While mortuary garments were not recovered from this specific burial, taphonomic evidence for wrapping bodies and use of ochre in the wrapping process has been reported at Zvejnieki (*61*). The presence of ochre on this axe and within this grave indicates a potential link between this tool and the processing of funerary garments; however, it remains impossible to rule out general hide-working activities (*79*). Either way, these findings help identify broader contexts for differences in manual activity between females with pendants and without pendants. Here, biomechanical results indicate a specialized pattern of manual activity among females buried with pendants that is consistent with hide-working. The analysis of funerary axes identifies a potential medium for these behaviors across the ethereal threshold that may be linked to hide-working within the settlement or for garments used in funerary rites.

In contrast, females without pendants have right and left humeral *I_x_*/*I_y_* values distributed slightly above 1.0. This is associated with similar *I_x_* values but slightly elevated *I_y_* values when compared to females with pendants. Similar contrasts were observed between groups that processed large terrestrial and aquatic mammals versus those where riverine fishing was emphasized: Females from communities that engaged in hunting of aquatic and terrestrial mammals had substantial *I_x_*/*I_y_* values in association with hide processing, while those from fishing villages had slightly elevated *I_x_*/*I_y_* values in relation to fishing behaviors (*40*, *45*). Fish processing tools at Zvejnieki included knives which were manufactured from ungulate ribs (*76*). Previous studies also report widespread female participation in net fishing, and these activities produced subtle increases in humeral *I_x_*/*I_y_* (*78*). In this sense, *I_x_*/*I_y_* values among females without pendants may be associated with specialized habitual activities that reflect less concentrated patterns of strain in the A-P plane than females with pendants. It is important to point out that the dichotomy between female and male behavior identified by this study does not erase female contributions to hunting. Divisions of labor between individuals estimated to be female and male are diverse among hunter-gatherer communities (*40*, *80*), and hunter-gatherers view behaviors such as immobilization, processing, and ritual interaction with the natural world as interconnected (*81*, *82*). Cross-sectional studies of female long bone diaphyses also point toward participation in strenuous activity (*39*, *40*, *83*).

Findings from this work add to the growing body of evidence for the emergence of social complexity in Mesolithic-Neolithic hunter-fisher-gatherers, specifically in relation to the dimensions reflected in mortuary practices (*5*, *7*, *8*, *13*, *61*). At Zvejnieki, biomechanical analyses differentiating humeral diaphyseal loading in relation to pendant usage builds on previous findings that report dietary distinctions in the same burials (*58*). Delineations in bone loading between individuals with pendants and without pendants support the interpretation that these two groups engaged in differentiated habitual activity: Males with pendants had humeral loading patterns consistent with unimanual projectile technology, while those without pendants experienced humeral loading patterns consistent with flexion and extension behaviors, particularly net fishing. Bone deposition indicates that diaphyseal loading associated with unimanual projectile use began in adolescence and finds further consistency with work that identifies differences in diet in relation to pendant usage before adulthood (*56*, *58*). In contrast, females with pendants appear to have engaged in hide processing, while females without pedants likely engaged with less intensive processing or net fishing. While the number of female remains included in this study is reduced, the magnitude of difference does support variation in manual behavior.

Previous studies argue that the emergence of institutional inequality is associated with occupational specialization (*24*, *84*). Ethnographic examples of hunter-gatherer social organization identify familial identities that differentiate into groups focused on fishing and hunting in Northern Japan and the Pacific Northwest Coast (*24*). These familial groups affiliate with deities linked to each ecosystem, which further included ownership of symbolic material goods, control over ceremonial events, and differential access to foodstuffs. Meat from terrestrial mammals is a high prestige item in terms of caloric return, symbolic authority, and cosmological status (*85*–*87*). However, select ethnographic examples point toward limited control over the production of resources in complex hunter-gatherers (*88*), and deeper inequalities relating to occupational specialization should be occasioned by evidence that these structures become embodied (*89*). While differences in diet and behavior typify mortuary patterning at Zvejnieki, little evidence for institutional control over resources is reported (*58*). Instead, results from this study point toward an emerging pattern of complexity where differences in mortuary treatment and diet are associated with delineations in habitual activity.

Differences in manual activity identified at Zvejnieki may reflect independent specialization, where goods are produced in the absence of a managerial class that controls the production and distribution of resources and/or derives power from these items (*21*). The utilization of grave goods to distinguish between individuals with varying occupational specializations may be associated with the materialization of ideology (*90*). Items such as grave goods facilitate symbolic communication (*91*), and the exchange of these items may create or reinforce horizontal or vertical relationships (*90*). This pattern of behavior distinguishes hunter-gatherer communities where memory becomes objectified through property and inheritance from those where communicative systems of memory are emphasized (*92*). Mortuary rituals and associated artifacts are commemorative practices achieved through repeated performance which are archaeological visible through redundancies (*93*). These performances produce and reproduce social memory (*94*). Variation in habitual activity and resource allocation between individuals with and without pendants at Zvejnieki suggest that the allocation of burial pendants represent important pathways through which memory becomes objectified, concentrated within specific occupational groups, and illustrates emerging layers of complexity in Mesolithic-Neolithic hunter-fisher-gatherer burial rites. Results from this study highlight the deeply transformative nature of hunter-gather social organization during the Mesolithic and emphasize the growing body of evidence that the Neolithic revolution reflects a broad adoption and modification of hunter-gatherer behavior. These findings support the growing body of literature that identifies the expanding social dimensions of hunter-gatherer behavior (*5*, *8*, *14*, *58*, *60*, *61*, *95*) and challengies the notion that hunter-gatherers lacked multidimensional social organization (*22*).

## MATERIALS AND METHODS

Human remains from approximately 330 burials containing at least 350 individuals were excavated between 1965 and 1977 (*6*) as well as 2005 and 2009 (*52*). Table 2 lists the sample sizes for female and male femora that were included in this work, while Table 3 lists the sample sizes for female and male humeri that were included in this work. All burials included in this work were assigned to cultural periods based on artifact chronologies and radiocarbon dates associated with the Middle Mesolithic and Late Neolithic occupation (*6*). Comparative cross-sectional properties were derived from the European database (*96*) that includes Early Upper Paleolithic, Late Upper Paleolithic, Mesolithic, and Neolithic remains (https://fae.johnshopkins.edu/chris-ruff/). These measurements provide valuable contextual information as patterns of mobility and engagement with terrain have been established according to detailed chronological, cultural, and ecological data (*96*). Table 4 lists the behavioral characterizations of these groups summarized by Holt *et al.* (*38*), with cultural affiliations and dates provided by Ruff (*96*).

**Table 4.** Context for European comparisons. Socioecological descriptions for the comparative groups included in this analysis.

Group	Mobility environment	Approximate dates^1^
Early Upper Paleolithic	High residential and logistical mobility; large geographic range; large game	33,000–26,000 BP
Late Upper Paleolithic	Reduced distance between residences; reduced logistical mobility; concentrated resources	22,000–11,000 BP
Mesolithic	Declining residential mobility; elevated logistical mobility; smaller game; larger settlements	8550–4000 BP
Neolithic	Reduced residential and logistical mobility; agriculture; ceramics; permanent villages	5350–2050 BP

Archaeological records of mortuary practices were used to identify individuals with and without pendants (*6*, *52*, *60*). Graves that include pendants were differentiated from those that did not include pendants. Under circumstances where backfill from later time periods was identified in a grave (*61*), those individuals were removed from the analysis due to uncertainties in the presence/absence of pendants within the burial (*58*).

Sex was estimated using morphological features of the skull and pelvis (*97*) as well as proteomics. Individuals used in the analysis of cross-sectional properties ranged in age from 18 to 55 years to control for changes in depositional surfaces during ontogeny and age-related bone loss. Age was estimated using long bone epiphyseal fusion, auricular surface morphology, pubic symphseal morphology, and tooth wear (*97*). Femora were scanned at 50% of length (*98*), and humeri were scanned at 35% of length measured from the distal end (*98*) using an SA+ portable PQCT scanner by Stratec (Pforzheim, Germany). Cross-sectional properties were calculated from the images using a macro provided by Stratec, and the software was tested daily using a phantom with known properties. Moments of area include TA, CA, and MA (Table 1). Second moments of area in this study include A-P (*I_x_*) and M-L (*I_y_*) humeral and femoral rigidity and overall humeral and femoral rigidity (*J*) (Table 1). Shape ratios were derived as the ratio of A-P relative to M-L rigidity for femora and humeri. Moments and second moments of area were compared for humeri between pendant groups to address complexities in interpretation of activity in the upper limb. Second moments of area were compared between groups for femora. Directional asymmetry for overall humeral rigidity was calculated as {(right − left)/[(right + left)/2]} ∙ 100.

Second moments of area were standardized by body mass * bone length^2^. Body mass was estimated using superior-inferior measurements of femoral and humeral head breadths (*99*, *100*). Comparative cross-sectional properties derived from the European database were collected using CT scans and molds of the external contour of the diaphyses (*38*). Cross-sectional properties estimated using these methods are comparable to those obtained from CT scans (*101*).

Owing to the uneven and often diminished sample sizes typical of bioarchaeological assemblages, comparisons relied on 95% credibility intervals and nonparametric resampling. Ninety-five percent credibility intervals for group means were computed to provide visual estimates of the distribution and uncertainty resulting from sample composition. These intervals were modeled with unknown means and variances using a conjugate normal-inverse-gamma model. Posterior draws of μ were generated by sampling σ^2^ from the inverse-gamma posterior and μ from the corresponding conditional normal posterior. Credibility intervals were defined by the 2.5th and 97.5th percentiles of these draws, using weakly informative hyperparameters (κ_0_ = 0.001; α_0_ = 0.001; β_0_ = 0.001·s^2^) and a prior mean centered on the group sample mean.

Comparisons of cross-sectional geometric properties (male pendant presence versus absence; females and males from Zvejnieki versus Paleolithic-Neolithic time periods) used Monte Carlo randomization of group mean differences over 10,000 permutations. This procedure generated a sampling distribution of mean differences under label exchangeability. The observed mean differences for each cross-sectional property $\left(∆={\bar{X}}_{\text{pendant}}-{\bar{X}}_{\text{none}};∆={\bar{X}}_{\text{Zvejnieki}}-{\bar{X}}_{\text{Comparative Group}}\right)$ was then plotted in relation to the randomized distributions of mean differences.

ROPEs within the sampling distribution were calculated to identify intervals within each distribution where the observed mean differences were negligible. Here, variables were grouped on the basis of meaning and scale, with areas (7% threshold), second moments of area (10% threshold), and diaphyseal shape ratios (3% threshold) forming independent groups. Thresholds were established in relation to biological sensitivity, scaling with body size, and measurement stability across variable classes. For directional asymmetry (%DA), thresholds for ROPE were set at 5, 10, and 15% differences in association with baseline, elevated, and strong signals of unilateral loading across time and subsistence category (*69*). ROPE widths were defined using class-specific proportional thresholds $\left[\beta_{v}^{\left(\%\right)}=p_{\text{class}}\times {\text{Reference}}_{v}\right]$ and, where available, multiples of the technical error of measurement $\left[\beta_{v}^{\left(\text{TEM}\right)}=k\times {\text{TEM}}_{v}\right]$. The final ROPE was defined as the larger of the two variables: $\beta_{v}=\text{max}\;\left[\beta^{\left(\%\right)},\beta^{\text{TEM}}\right].$ These computations were performed in the R programming environment using author input and ChatGPT-5.2 to edit and generate code. All code and data for the Zvejnieki cross-sectional properties are available via GitHub: https://github.com/dht1979/Zvejnieki-Occupational-Differentiation.

## Supplementary Materials

### This PDF file includes:

Provenance Statement
