# Supplementary material for: Biomechanical evidence for occupational specialization in Mesolithic-Neolithic hunter-gatherers from Zvejnieki, Latvia
Source: Sci Adv. 2026 Jun 12;12(24):eaed3371. doi: 10.1126/sciadv.aed3371 (PMC13262614; doi:10.1126/sciadv.aed3371)
Supplement: Supplementary file 1 — Provenance Statement [file sciadv.aed3371_sm.pdf]

Science Advances
AAAS

Supplementary Materials for

Biomechanical evidence for occupational specialization in Mesolithic-Neolithic hunter-gatherers from Zvejnieki, Latvia

Daniel H. Temple *et al.*

Corresponding author: Daniel H. Temple, dtemple3@gmu.edu

Sci. Adv. **12**, eaed3371 (2026)
DOI: 10.1126/sciadv.aed3371

This PDF file includes:

Provenance Statement

Provenance Statement

The Zvejnieki archaeological site is located in the Vecate Village of the Valmiera District on the Astrumi State Farm within the Zvejnieki farmstead. The site lies on the southeastern portion of a gravel ridge (drumlin). Human remains were noted at Zvejnieki by George Sievers in 1874, though many graves were disturbed. In 1926 Rauls Šnore reported human remains at the site to the Board of Monuments. Ochre stained human remains were found in 1964 during a gravel excavation. This was reported to Laimonis Liepniks from the Valmiera Regional Museum. Janis Apals evaluated the site and identified multiple graves in the profile of the gravel pit, one with traces of ochre. Trial excavations were completed by the Institute of History of the Academy of Sciences of the Latvian SSR. The site was systematically excavated in 1965, 1966, 1968, 1970, and 1971 by Francis Zagorskis and Igla Zagorska. These efforts uncovered 315 burials dating from the Middle Mesolithic to the Neolithic. Lars Larsson and colleagues excavated an additional 26 individuals between 2005 and 2009. Remains used in this study have been assigned approximate cultural designations based on artifact association (6), with radiocarbon dating placing them into cultural categories such as Mesolithic and Neolithic (56-58). Human remains from the Zvejnieki site are now curated by the Institute for Latvian History, Faculty of Humanities, University of Latvia. Remains from Zvejnieki may be accessed with the expressed permission of the Institute for Latvian History, Faculty of Humanities, University of Latvia. Two individuals affiliated with this institution (G.Z. and I.Z.) and one of the original excavators (I.Z.) are co-authors on this paper.

In Latvia, archaeological and anthropological research procedures are regulated by Cabinet of Ministers Regulations: Regulations, Articles 35, 38. According to these regulations, archaeological research includes archaeological excavations, research of archaeological materials, archaeological monitoring, and archaeological survey, if it requires intervention in a cultural monument. There is no special legislation in Latvia on research of anthropological material.
